# Serous maculopathy with absence of retinal pigment epithelium (SMARPE) associated with large drusen

**DOI:** 10.1186/s40942-024-00529-5

**Published:** 2024-01-22

**Authors:** Luiz H. Lima, João Pedro Romero Braga, Gustavo B. Melo, Wener P. Cella, Adam S. L. Brandão, Rodrigo L. Meirelles, Claudio Zett, Francyne V. R. Cyrino, Rodrigo Jorge

**Affiliations:** 1 Department of Ophthalmology, Federal University of São Paulo (UNIFESP), Rua Botucatu, 821, Vila Clementino, São Paulo, 04023-062 Brazil; 2 Division of Ophthalmology, Ribeirão Preto Medical School, University of São Paulo, Ribeirão Preto, Brazil; 3 Hospital de Referência Oftalmológica, São Luís, Maranhão Brazil; 4 Pontificia Universidad Católica de Valparaíso, Valparaíso, Chile

**Keywords:** Age-related macular degeneration, Large drusen, Multimodal imaging, Serous maculopathy with absence of retinal pigment epithelium (SMARPE)

## Abstract

**Purpose:**

To describe the association of serous maculopathy with absence of retinal pigment epithelium (SMARPE) and large drusen in patients with non-neovascular age-related macular degeneration (AMD).

**Methods:**

A retrospective study of ophthalmic examination and multimodal imaging data of individuals with SMARPE and large drusen observed over a period of 12-month was accomplished. SMARPE was defined as subretinal accumulation of fluid within the macular area due to retinal pigment epithelium (RPE) aperture. Large drusen were identified by the presence of sub-RPE deposits using multimodal imaging analysis (color fundus photography, fundus autofluorescence, and spectral-domain optical coherence tomography).

**Results:**

Twelve eyes of 7 white patients with a mean age of 77 years were observed to have SMARPE associated with large drusen. The median visual acuity was 20/100. Bilateral SMARPE lesions were observed in 71% of study patients. All SMARPE lesions were hypoautofluorescent, located in the subretinal space between the RPE and the ellipsoid zone, and presented as complete or incomplete RPE apertures associated with subretinal fluid. The SMARPE in this study had coincident multimodal imaging features as the SMARPE described in other reports in the literature.

**Conclusions:**

Bilateral SMARPE can occur in association with typical AMD large drusen. Anomalisms resulting in drusen biogenesis or mechanisms that act alongside to these may be related to SMARPE development.

The clinical spectrum of chorioretinal diseases that may present with subretinal fluid (SRF) or a SRF-like feature in the macula is broad and includes several disorders categories, such as retinal vascular disorders, genetic diseases, hematological malignancies, ocular developmental aberrations, neovascular disorders, medication-related and toxicity-related conditions, inflammatory diseases, ocular tumors, paraneoplastic syndromes, rhegmatogenous and tractional retinal detachment, vitelliform lesions and serous maculopathy with absence of retinal pigment epithelium (SMARPE) [1, 2]. 

SMARPE refers to the absence of the retinal pigment epithelium (RPE) in the macula associated with SRF [2]. Querques et al. described SMARPE, for the first time in 2016, as RPE apertures in the evolution of avascular pigment epithelium detachment (PED) secondary to age-related macular degeneration (AMD) [3]. Previously, Goldstein et al. reported similar RPE defects named as “blow-outs” of the RPE in central serous chorioretinopathy [4]. Differently from the RPE microrips that represent single microtears associated with PED due to macular neovascularization (MNV), SMARPE develops in AMD eyes without an evident MNV. The SMARPE patients present with atypical and central RPE defects which are not due to RPE tears or geographic atrophy [2–5]. 

As there is no evidence of MNV, the focal RPE loss and, therefore, its failure to pump ions and fluid out of the subretinal space may be the intrinsic cause of SRF accretion in SMARPE [2]. The multimodal imaging of SMARPE highlights the RPE loss with corresponding hypoautofluorescence on fundus autofluorescence (FAF). On fluorescein angiography (FA), window defect associated with gradual pooling into the subretinal space due to the dysfunctional RPE is observed. Spectral-domain optical coherence tomography (SD-OCT) demonstrates the lack of the segment of the hyperreflective line that represents the RPE [2, 3, 5]. 

In the literature, there are only a few case reports of SMARPE, and all of them are unilateral. Using multimodal imaging, we report an unique case series of bilateral SMARPE associated with large drusen in patients with non-neovascular AMD.

## Methods

This study followed the Tenets of the Declaration of Helsinki. Institutional Review Board of the Division of Ophthalmology, Ribeirão Preto Medical School, University of São Paulo, Ribeirão Preto, Brazil (Institutional Review Board number: 6.199.272) approval was obtained for this retrospective data analysis.

The imaging data and medical records of a consecutive series of non-neovascular AMD patients with SMARPE associated with large drusen presenting between January 2023 and June 2023 were retrospectively reviewed from tertiary referring institutions: (1) Federal University of São Paulo, São Paulo, Brazil; (2) University of São Paulo, Ribeirão Preto, Brazil; (3) Hospital de Referência Oftalmológica, São Luís, Brazil; and (4) Pontificia Universidad Católica de Valparaíso, Valparaíso, Chile.

All study patients underwent a complete ophthalmologic examination, including color photography, FA, FAF and SD-OCT. FAF images were obtained using either the confocal scanning laser ophthalmoscope (Heidelberg Retina Angiograph, HRA2; Heidelberg Engineering, Heidelberg, Germany) or a fundus camera (Topcon Medical Systems, Tokyo, Japan). The location of SMARPE and large drusen was studied by analyzing the available SD-OCT data. The SD-OCT scans were obtained with the DRI Triton OCT (Topcon Medical Systems, Tokyo, Japan) in 4 patients, with the Cirrus SD-OCT (Carl Zeiss Meditec, Inc, Dublin, CA) in 2 patients, and with the Heidelberg Spectralis (Heidelberg Engineering GmbH, Heidelberg, Germany) in 1 patient.

The large drusen were identified by color fundus photograph and SD-OCT. The size scale grading in which drusen are classified as small (< 63 μm), intermediate (> 63–125 μm), or large (> 125 μm) was used [6]. The size of drusen was determined by the caliper available on the imaging software (Topcon TRC-50XF, Topcon Medical Systems, Tokyo, Japan). Large drusen were diagnosed if there were dome-shaped eminences with measurement, in diameter, between 125 μm and 1,000 μm and with corresponding hyperreflective material between the RPE and Bruch membrane on SD-OCT [6]. SMARPE lesion was considered to be present if there was SRF collection associated with a striking absence of RPE within the macular area. Only eyes diagnosed with SMARPE and large drusen were included in this case series. Patients that had a history of SRF related to other ocular, including cuticular drusen and subretinal drusenoid deposits (SDD), or systemic disorders were excluded. In addition, eyes having large drusen in association with any signs of MNV were not included in this study.

MNV was excluded by the absence of any evidence of signs of leakage on the mid or late FA stages. The absence of cuticular drusen and SDD was defined by the lack of small drusen with a presentation of “stars in the sky” on FA and as less than five drusenoid lesions above the RPE on SD-OCT, respectively [6, 7]. 

The statistical analysis included descriptive statistics for demographic data and main clinical features. Data were analyzed with the Statistical Package for the Social Sciences (IBM, Chicago, IL).

## Results

We analyzed 14 eyes of 7 non-neovascular AMD patients with SMARPE and large drusen. All study patients were white, and there were 5 men and 2 women. The mean age was 77 years (range: 69–83 years). The best-corrected visual acuity at the time the SMARPE was first imaged ranged from 20/30 to counting fingers (median visual acuity: 20/100). The study eyes had a mean follow-up of 6 months (range: 2–14 months). The mean visual acuity at the most recent examination was 20/200 (median: 20/250). In 3 eyes (21%), visual acuity decreased over the follow-up period. Two patients did not present to follow-up examination. In all study eyes, large drusen were observed as convex collections of hyperreflective material located in the sub-RPE space. All study patients had several large drusen within the macular area. The SMARPEs were observed in 12 eyes, and presented as circumscribed RPE discontinuity within the macular area.

**Fig. 1:**
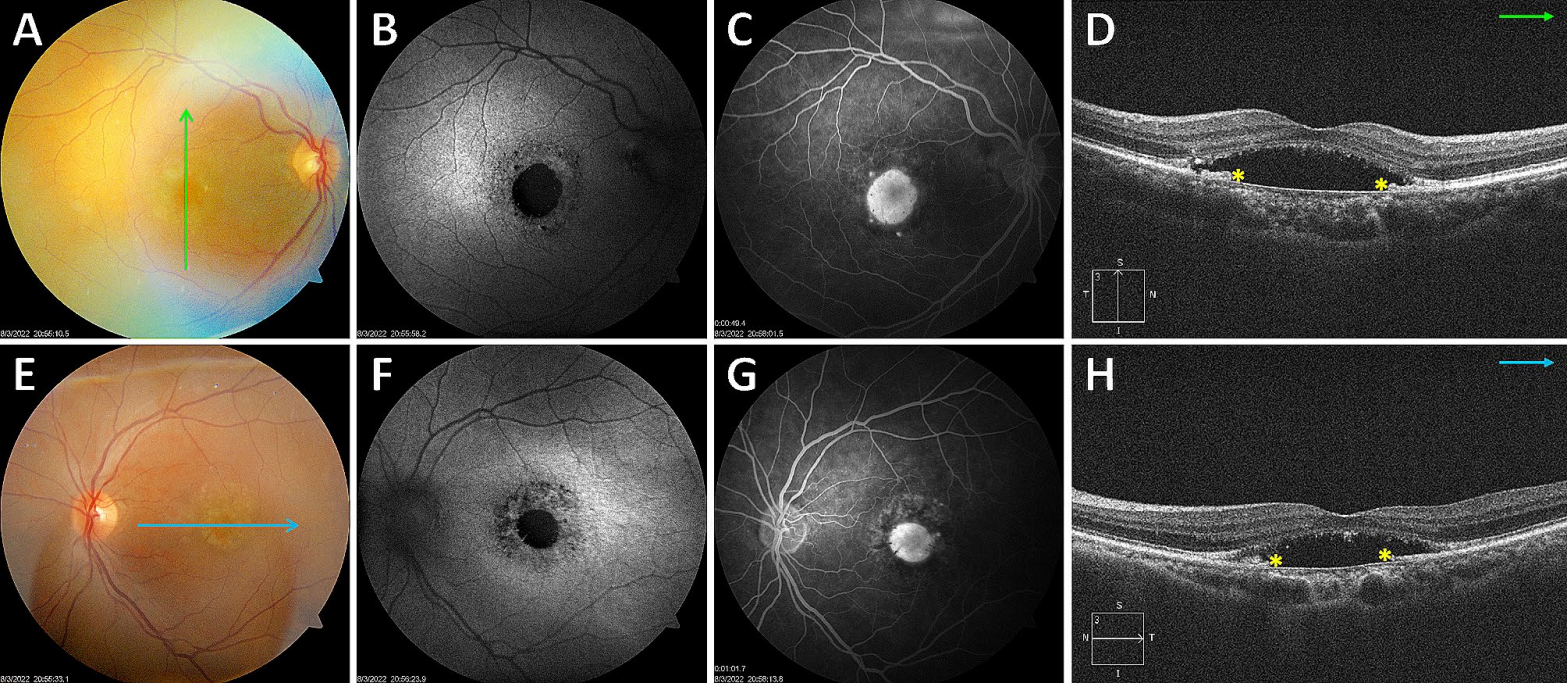
Multimodal imaging in a 75-year-old woman **(Case # 1)** presenting with bilateral serous maculopathy with absence of retinal pigment epithelium (SMARPE). Visual acuity was 20/150 in the right eye and 20/200 in the left eye. Color fundus photograph of both eyes (**A** and **E**) shows several typical age-related macular degeneration (AMD) large drusen and retinal pigment epithelium (RPE) atrophy within the macular area, with correspondent hypoautofluorescence on fundus autofluorescence (FAF) (**B** and **F**), and hyperfluorescence due to window defect on fluorescein angiography (FA) (**C** and **G**). Cross-sectional spectral-domain optical coherence tomography (SD-OCT) of both eyes (**D** and **H**) depicts the incomplete type **(asterisks)** of SMARPE (there is part of the RPE between the SRF edges) in the macular area. An intact ellipsoid zone (EZ) and external limiting membrane (ELM) overlying the RPE defect are observed in both eyes

In all SMARPE eyes, FAF demonstrated corresponding hypoautofluorescence at the SMARPE topography on color fundus. FA showed early hyperfluorescence corresponding to the RPE defect along with pooling in the late imaging phases. On SD-OCT, SMARPE was characterized by a round RPE discontinuity associated with SRF accumulation within the macular area (Fig. 1). RPE retraction or rippling was not found at the sites of SMARPE, differently from RPE rips or tears. In our series, only 1 eye had an avascular PED. Preserved ellipsoid zone (EZ) and external limiting membrane (ELM) overlying the RPE defect was depicted in all included eyes. Hyporeflective spaces located between the RPE and the EZ and consistent with SRF were detected in 10 eyes (71%). In 4 patients, the fellow eye did present with large drusen solely or retinal and RPE atrophy. Hyperreflective material within the SRF was detected in 8 eyes, and intraretinal hyperreflective foci were found in 3 eyes. All study eyes had large drusen within 500 μm of SMARPE, and they did not show hyperreflectivity with near infra-red reflectance imaging on the scanning laser ophthalmoscope.

**Fig. 2:**
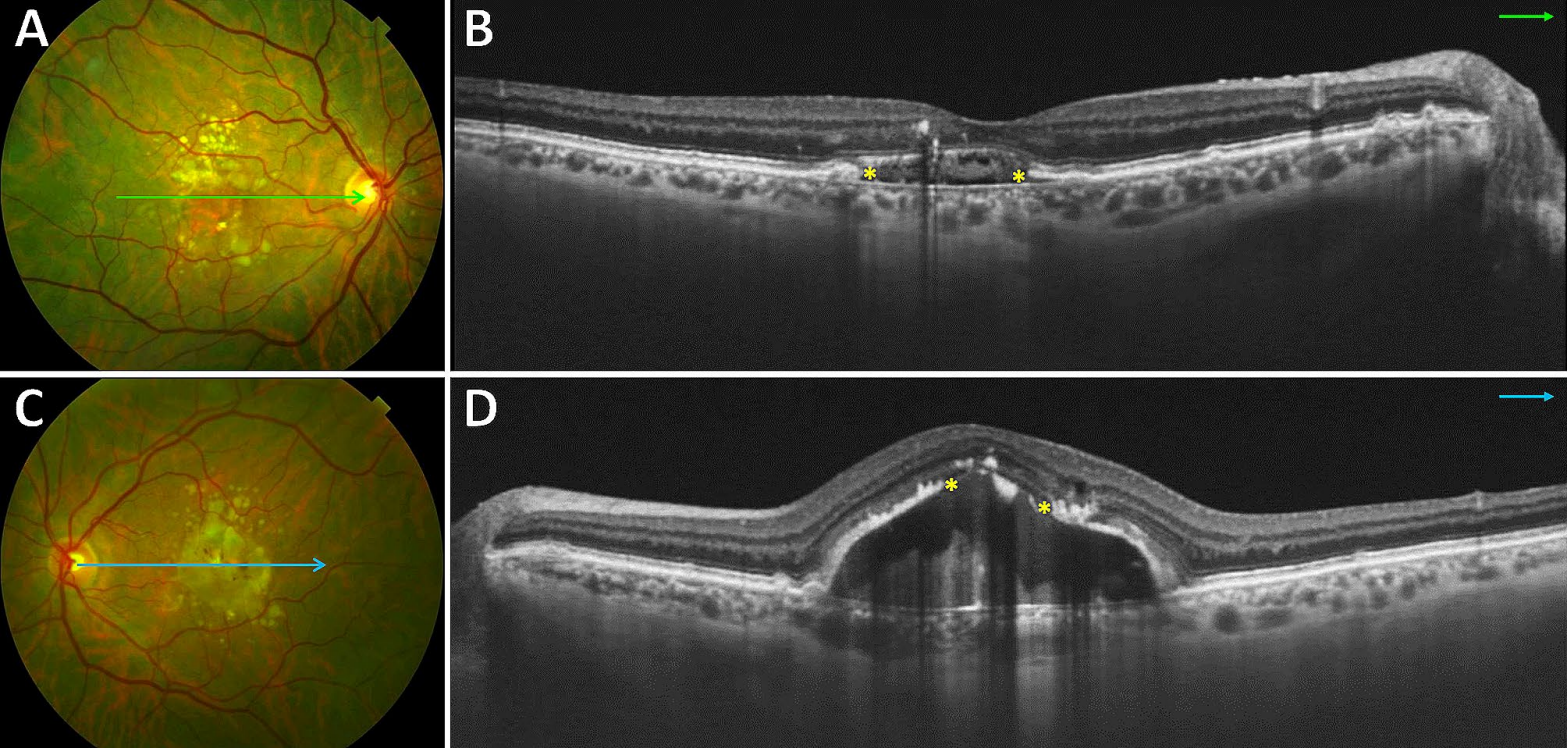
Multimodal imaging in a 69-year-old man **(Case #3)** with bilateral SMARPE. Visual acuity was 20/80 in the right eye and 20/60 in the left eye (**A** and **C**). Color fundus photograph of both eyes demonstrates RPE atrophy surrounded by an agglomerate of AMD large drusen within the macular area. (**B** and **D**). Cross-sectional SD-OCT shows complete and incomplete types **(asterisks)** of SMARPE in the right and left eyes, respectively

**Fig. 3:**
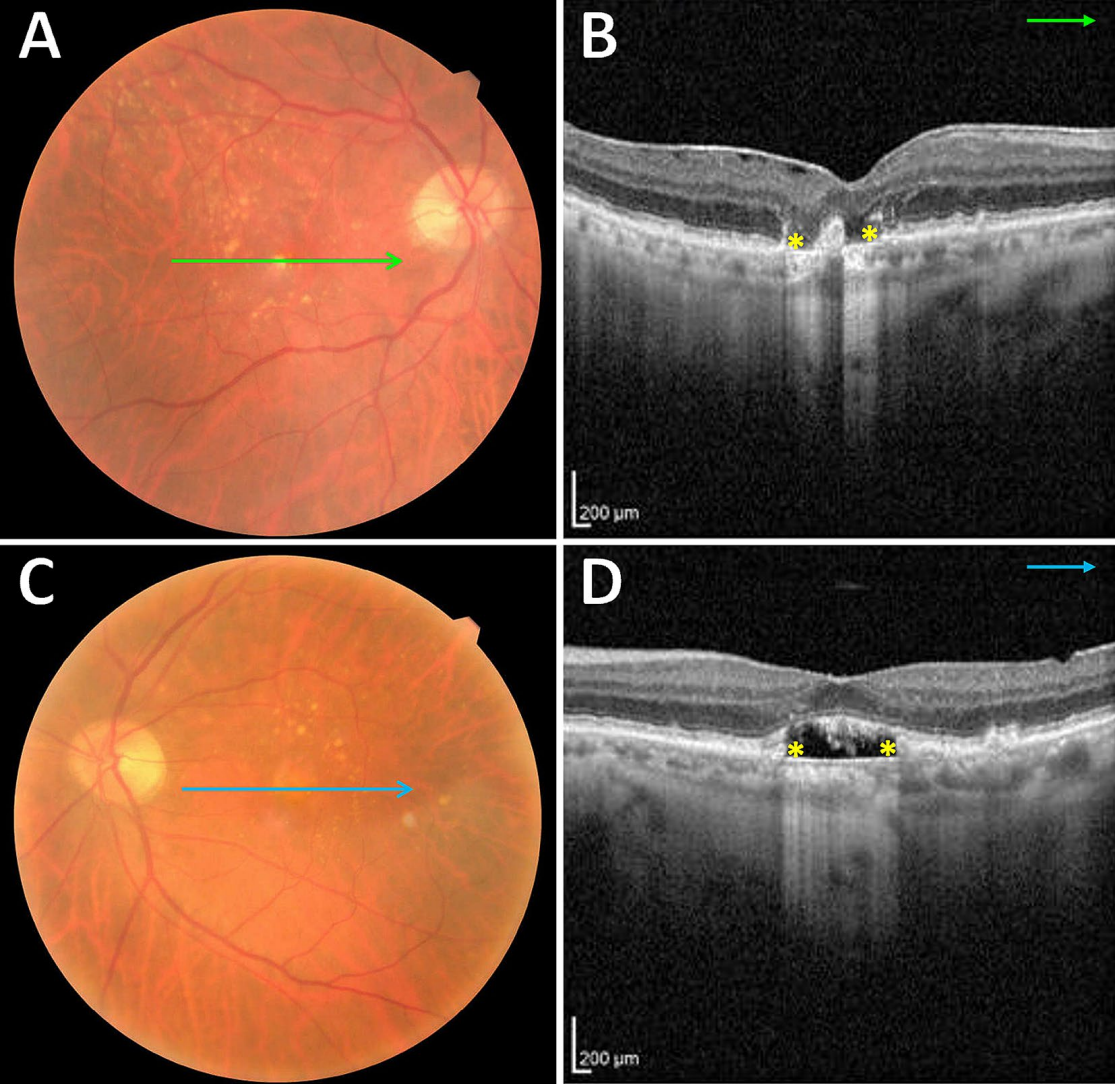
Multimodal imaging in an 80-year-old man **(Case #7)** with unilateral SMARPE. Visual acuity was 20/80 in the right eye and 20/50 in the left eye. (**A** and **C**). Color fundus photograph of both eyes demonstrates several typical AMD large drusen within the macular area. (**B** and **D**). Cross-sectional SD-OCT shows a complete RPE and outer retinal atrophy (cRORA) **(asterisks)** in the right eye, and a complete type **(asterisks)** of SMARPE in the left eye

Five patients (71%) were found to have bilateral SMARPEs (Fig. 2). Based on the SRF edges and the hyperreflective line that represents the RPE on OCT, we classified SMARPE into two types: complete (total absence of RPE between the SRF edges) and incomplete (presence of RPE between the SRF edges). Complete and incomplete SMARPE were observed in 4 (33%), and 8 eyes (67%), respectively. A larger amount of SRF accretion was observed in the eyes with complete type in comparison with the incomplete type of SMARPE. The unilateral cases (Cases #5 and #7) presented with a SMARPE lesion in one eye and with a complete RPE and outer retinal atrophy (cRORA) in the fellow eye (Fig. 3).

None of the included eyes demonstrated evidence of MNV during the study period. Despite the absence of a definite MNV, 10 eyes with SRF received intravitreal anti-VEGF injections without improvement of visual acuity. In Case #5, micropulse laser was performed in the eye with SMARPE lesion after 3 injections of aflibercept, but also without visual acuity improvement. Persistence of SRF was found in all study eyes during the follow-up period, and, therefore, did not respond to anti-VEGF treatment.

## Discussion

In this case series, SMARPEs associated with large drusen were observed in 12 eyes of 7 patients. None of the study participants had retinal or systemic disorders related to SRF or hyperreflective material accumulation such as vitelliform macular dystrophy, tractional maculopathies, central serous choroidopathy, adult-onset vitelliform dystrophy, persistent SRF following retinal surgery, choroidal tumors, paraneoplastic diseases, and acute exudative polymorphous vitelliform maculopathy [6, 8–13]. None of the cases was associated with RPE tear, laser treatment, hemorrhage or retinal exudate neither showed resolution or decreased amount of SMARPE lesion. Also, none of the study patients developed MNV during the mean follow-up of 6 months.

Typical RPE unifocal defects associated with SRF within the macular area and related to visual loss were observed in all study patients. SRF and hyperreflective material accretion were found in 71% and 57% of study eyes, respectively. The material accumulation found in the subretinal space is probably related to an impaired RPE phagocytosis [9, 14]. Possible pathological mechanisms could explain this abnormal RPE phagocytosis. The SRF found in SMARPE may interrupt the apposition between the RPE and photoreceptor outer segments as observed in the study eyes. This loss of apposition can interfere with the phagocytosis of shed outer segments, resulting in the material accumulation in the subretinal space. The second possibility is that the lack of RPE within the macular area in SMARPE patients may result in focal inability of RPE cells to phagocytize the photoreceptor outer segments [15, 16]. 

In contrast with the other SMARPE cases reported in the literature which are unilateral, the majority (71%) of our cases were bilateral. It could be explained by the different SMARPE lesion development mechanisms. In the current series, all lesions were associated with AMD large drusen that are typically bilateral. Incomplete lesions (67%) occurred more commonly than the complete lesions (33%), with a greater amount of SRF accumulation in the eyes with the complete type of SMARPE. Probably, this is related to a larger extension of RPE defect in the complete type resulting in a greater area of impaired RPE function. In this series, at presentation, cRORA was found in the fellow eye of both unilateral SMARPE cases (Cases #5 and #7), and it may represent a late evolution of the SMARPE lesion. Another possibility is that SMARPE could be a variation of geographic atrophy that does not have the ability to drain the SRF. In this setting, the frequency of SMARPE is probably underestimated in AMD patients with geographic atrophy.

The dysfunction of RPE has been implied in drusen development [6], and similar abnormalities may influence the accumulation of SRF in patients with SMARPE. The material forming drusen (basal linear deposit) would interfere in the RPE function, and, therefore, contribute to SRF accumulation. The defective RPE, in conjunction with inflammation, has been associated with drusen biogenesis [6]. Although the process of drusen formation is still unknown, some reports have shown that the complement system [6, 17, 18] and dendritic cells [6, 19, 20] may represent important players in inflammation associated with the development of drusen. The iatric reaction to a Bruch membrane filled with lipoproteins is another conjecture for drusen development [6, 21, 22]. The exact pathological process of the SRF associated with large drusen observed in the present SMARPE cases is unknown, but it could be due to the focal lack of the RPE, and, therefore, its inability to pump ions and fluid out of the subretinal space.

This study has some limitations as the small number of cases and its descriptive nature that allows only speculative enlightenments regarding the precise pathogenic mechanism that explains the association of large drusen and SMARPE. Additionally, the prevalence of SMARPE in non-neovascular AMD patients need to be measured in an appropriate research investigation. Regardless this, our report of SMARPE in conjunction with large drusen may contribute to establish potential hypotheses that explain such imaging association.

## Data Availability

The datasets used and/or analysed during the current study are available from the corresponding author on reasonable request.
